# Software Changes and Technical Failures in Clinical Trials of Digital Interventions Used Alongside Pharmacotherapy: Narrative Review of Regulatory Guidance and Published Evidence

**DOI:** 10.3390/healthcare14213604

**Published:** 2026-10-24

**Authors:** Inyoung Hwang, Ki Young Huh, Kyung-Sang Yu, Sang Won Lee

**Affiliations:** 1 Department of Clinical Pharmacology and Therapeutics, Hanyang University Seoul Hospital, Seoul 04763, Republic of Korea; isoleucine@hanyang.ac.kr; 2 Department of Pharmacology, Hanyang University College of Medicine, Seoul 04763, Republic of Korea; 3 Department of Clinical Pharmacology and Therapeutics, Seoul National University College of Medicine and Hospital, Seoul 03080, Republic of Korea; zealot648@snu.ac.kr (K.Y.H.); ksyu@snu.ac.kr (K.-S.Y.)

**Keywords:** digital therapeutics, pharmacotherapy, software changes, technical failures, clinical trial methodology

## Abstract

**Background/Objectives**: Software changes and technical failures can alter the treatment delivered by a digital intervention or the data used to evaluate it. In trials alongside pharmacotherapy, medication use and replacement care may also affect the comparison. This review examined which functions software events affected and whether the available evidence supported the intended treatment comparison. **Methods**: A focused narrative review combined 22 public guidance and related documents, two product records, and 67 academic publications selected from PubMed searches completed on 10 September 2026. Cases were compared by the function involved, reported response, and information needed to assess remaining uncertainty. **Results**: CureApp’s assessment found no health effects from reported malfunctions but did not quantify interrupted treatment or lost endpoint observations. Spark incorporated software version into pooled analyses but did not report separate treatment effects for each version. In Quitting Matters, incomplete recording affected a primary engagement outcome, and retaining partial records did not establish comparable capture between groups. These examples distinguish evidence that an event occurred, evidence that it was addressed, and evidence about its effect on the comparison. They do not estimate the frequency of software events or their causal effects on efficacy. **Conclusions**: Appraisal requires assessment of both intervention integrity and data integrity. Investigators should link affected functions and event periods to treatment delivery, medication use, replacement care, and outcome records, then evaluate whether the analysis addresses the intended comparison. Repair or absence of observed harm alone cannot establish that the intended treatment comparison remains supported.

## 1. Introduction

Digital therapeutics (DTx) can provide behavioral or cognitive treatment alongside pharmacotherapy [1]. CureApp, for example, was evaluated within standard smoking-cessation care in which most participants received varenicline or nicotine patches [2], the Therapeutic Education System (TES) provided computerized behavioral treatment to patients receiving buprenorphine [3], and CT-152 was tested in the MIRAI remote trial during antidepressant monotherapy [4,5]. Although the conditions differ, these trials ask the same question: whether adding a digital intervention improves outcomes beyond those achieved with the medication and other care that the control group also receives.

Software changes and technical failures during a trial can make this added benefit harder to interpret. Changes in therapeutic content or access may alter the treatment participants receive, and errors in usage records may obscure how much the intervention was actually used. The comparator matters as well, because a control application may itself provide monitoring, education, or clinical contact alongside pharmacotherapy [1,6]. An event in either arm therefore has to be judged against what the affected application does and the treatment comparison specified in the protocol.

The Pharmaceuticals and Medical Devices Agency (PMDA) review of CureApp shows why this matters. PMDA documented malfunctions in both trial groups and concluded that none had affected participants’ health [7]. This assessment addressed reported health effects. For the efficacy comparison, however, two further questions arise: whether participants could continue to use the digital treatment as planned, and whether the outcome data were complete.

Existing guidance offers a starting point. US Food and Drug Administration (FDA) guidance covers validation of digital health technologies, software updates, and comparability of measurements [8], and European Medicines Agency (EMA) guidance deals with the reliability of computerized systems and electronic trial data [9]. The International Council for Harmonisation (ICH) E9(R1) addendum provides a framework for defining the treatment effect of interest, or estimand, and for handling events that affect its interpretation [10]. Korean Ministry of Food and Drug Safety (MFDS) guidance on digitally enabled data collection explicitly considers how device failures can compromise trial data and influence clinical decisions [11].

Previous reviews have examined the regulatory acceptance of digital endpoints, statistical issues with digital outcome measures, and adherence information in trials of software-based interventions [12,13,14,15]. Applying these principles to a reported software event requires linking the event and its response to the treatment delivered and the observations used in the planned comparison. The present review examines software changes and technical failures in trials of digital therapeutics and patient-facing medication guidance used alongside pharmacotherapy. Research question 1 (RQ1) asks which functions an event affected, including treatment delivery, outcome measurement, medication use, and replacement care. Research question 2 (RQ2) asks whether the available data and analysis still support the intended treatment comparison.

## 2. Materials and Methods

### 2.1. Review Design and Scope

The researchers conducted a focused narrative review of public guidance and published evidence. This design was chosen because the question required interpretation across sources with different purposes, including trial reports, regulatory assessments, recall notices, and methodological guidance. The objective was to compare what documented events and responses establish about treatment and measurement, rather than to map all relevant studies or estimate event frequency. A systematic or scoping review would address a different objective of exhaustive retrieval or evidence mapping. Sources were selected purposively to illustrate distinct functions, event types, and responses. Records of actual events were distinguished from literature explaining treatment pathways, measurement, reporting, or statistical interpretation.

In this review, software changes are documented modifications to software content, logic, configuration, or version. Technical failures are documented problems in the operation, access, transmission, processing, or recording functions of a digital system. A device deficiency is not assumed to be a software-code defect unless the source identifies that cause. Digital interventions used alongside pharmacotherapy are patient-facing digital treatments or medication-guidance functions delivered in a care pathway with required or documented medication use. Medication use in only a subset is identified as such. Measurement-only and supportive-care systems provide explicitly identified context. Clinical co-use does not by itself establish the Korean statutory category of a digital convergence drug or the US combination-product category [16,17]. A software event denotes either a software change or a technical failure. Its classification as an adverse event or an ICH E9(R1) intercurrent event requires a separate assessment.

Treatment delivery means the availability and provision of the intended digital content, sessions, or therapeutic outputs. Outcome measurement includes acquiring, transferring, processing, and deriving observations used for evaluation, including engagement measures when specified as trial outcomes. Medication use concerns the prescribed and actual drug regimen, including initiation, dose, adherence, interruption, and discontinuation. Replacement care is care provided to substitute for or compensate for an affected digital function. The intended treatment comparison is the protocol-defined contrast between treatment strategies under their specified medication and comparator-care conditions. Safety findings concern reported harm or absence of observed harm. Repairs are technical actions intended to restore or correct system operation. Neither term implies that lost treatment or observations have been recovered.

### 2.2. Sources, Searches, and Selection Records

PubMed was searched through the National Center for Biotechnology Information (NCBI) E-utilities on 8–10 September 2026 without publication-date or language restrictions. Topic, product-name, and trial-name searches covered digital treatment alongside pharmacotherapy, software events, reporting, validation, digital endpoints, missing data, estimands, and named products or trials. Records retrieved only by DOI, exact-title, or author-specific lookups were outside this search frame. Supplementary Method S1 gives the search components and the yields reconstructed from the archived results. Public US, European Union (EU), Korean, and ICH documents available through 10 September 2026 were identified through official websites and cross-references between documents (Supplementary Method S2), and product-specific assessments and recall records were kept separate from general guidance.

The searches yielded 1093 unique PubMed identifiers. I.H. screened the titles and available abstracts of all records, consulting full texts when needed to judge relevance. Records were prioritized for detailed examination if they could provide evidence on an actual software event and its handling, clarify the digital function and medication pathway, or contribute relevant methodological or regulatory principles. S.W.L. reviewed the 77 candidate records and confirmed the selection decisions. Supplementary Method S3 describes the selection criteria.

**Figure 1:**
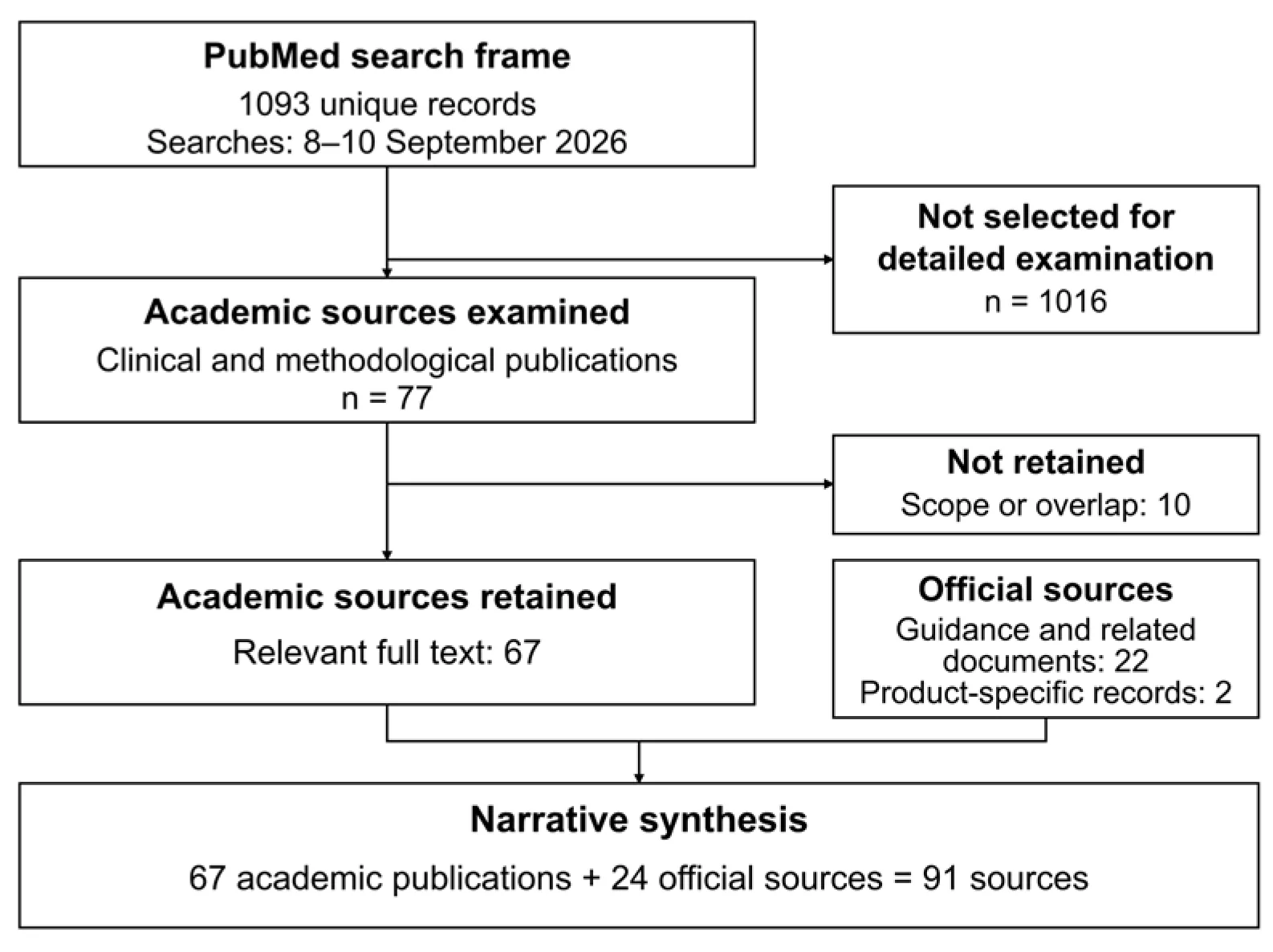
Selection of sources for the narrative synthesis. The diagram summarizes the search and selection records. The PubMed search frame yielded 1093 records, from which 67 academic publications were retained. The 24 official sources comprise 22 guidance and related documents and two product records. These counts describe source selection and are not denominators for software-event frequency.

Of the 77 records examined in detail, 67 were retained, and ten were not retained because of peripheral relevance or overlapping contributions. The other 1016 records were not selected. All 67 retained publications are cited in this article and listed with their reference numbers in the Supplementary Data. The review also included 22 public regulatory, agency, legal, or ICH documents and two product-specific records. Together, these 91 sources formed the narrative synthesis shown in Figure 1.

### 2.3. Eligibility Criteria and Access to Sources

Eligible sources required identifiable authorship or an issuing authority, a traceable publication record or document version, and accessible English or Korean text sufficient to evaluate the information used. Eligible guidance dealt with trial conduct, software change control, validation, reporting, data integrity, or interpretation of treatment effects, and trial reports had to describe the digital component’s function and the medication context. Implementation studies and postmarketing records served as contextual evidence and were interpreted in light of their settings. Promotional material and sources unrelated to the review question were not used as regulatory evidence, redundant document versions were counted once, and only published literature and publicly available official documents were used.

Relevant full texts were available for all 67 retained academic publications, although separate supplements, image-only material, and technical records were not always accessible. For CureApp, PMDA’s official English translation of the review report was used.

### 2.4. Extraction, Verification, and Synthesis

For official documents, the researchers extracted the issuing authority, legal status, scope, date, and relevant sections. For clinical sources, extraction recorded the digital function, medication regimen, comparator, event setting, function involved, documented consequences, and reported response (Supplementary Method S4). Implemented actions were separated from recommendations and from responses the source did not describe. Case appraisal first identified affected functions (RQ1), then considered what the reported response resolved and what information or analysis was needed to assess the intended comparison (RQ2). Supplementary Method S5 describes these questions. I.H. reviewed the retained evidence. Bibliographic details were checked against PubMed and Crossref where available, and official references against agency or legal sources.

Clinical importance was appraised qualitatively from the function involved, duration and extent of the event, participants and trial groups affected, reported treatment or measurement consequences, and recovery. The appraisal distinguished observed consequences from plausible but unconfirmed consequences. A common severity scale was not applied because the sources used different denominators and provided different levels of detail. An event with unreported consequences was treated as uncertain, not as clinically minor. No causal effect of an event or pooled event rate was estimated.

## 3. Results

### 3.1. Public Guidance and Its Provisions for Software Events

The 22 public documents covered the intended digital function, validation, change control, data traceability, and interpretation of the treatment effect. Table 1 summarizes provisions relevant to identifying affected functions (RQ1) and assessing the evidence supporting the comparison (RQ2). These are complementary contributions across documents, not two requirements imposed by every source. Supplementary Method S2 describes the official-source searches and selection. Legislation and regulations impose obligations within their scope. Agency guidance, ICH guidelines, draft guidance, and technical discussion papers have different status and should be interpreted accordingly.

Sources are cited in each row. Provisions concerning function, intended use, and change impact inform RQ1. Validation, traceability, measurement comparability, and treatment-effect principles inform RQ2 within each document’s stated scope and legal status.

The FDA guidance on digital health technologies deals with updates during a clinical investigation, comparison of measurements before and after a change, and the handling of those measurements in the analysis [8]. The EMA guideline on computerized systems and ICH good clinical practice set expectations for validated systems, data integrity, access control, and traceable changes [9,18,19], and FDA final guidance on electronic records (October 2024, Revision 1) covers the reliability and management of electronic systems and records [28]. Together, these documents expect a change to be evaluated and the affected records to remain usable, beyond documenting the event itself.

The MFDS guideline on data collection, issued on 24 December 2025, addresses the review question directly. It discusses the rationale for device selection, software version management, update procedures, data flows, data-management planning, usability, and continuity arrangements, and it describes how a device failure could produce unreliable glucose data that affect both the trial analysis and insulin-related clinical care [11], an example in which measurement and medication use are affected together. The April 2026 MFDS guideline on digital-device trials similarly covers the reliability of remote data, implications for the primary endpoint, missing data, and recovery from network failures or data loss [26].

The September 2026 MFDS guideline on protocols for digital therapeutics in nicotine use disorder specifies the content of the trial plan, including the treatment and comparator descriptions and the efficacy evaluation [25]. It gives indication-specific context for CureApp without applying retrospectively to the Japanese trial. The July 2026 authorization regulation and the guidance on change-management plans for artificial intelligence (AI)-enabled digital medical devices (June 2026), software validation (July 2026), and management-system certification (August 2026) concern other stages of the product lifecycle [27,29,30,31].

The applicable procedures depend on whether a drug or a device is under investigation. In the United States, drug protocol amendments fall under 21 CFR 312.30 and changes to device investigations fall under 21 CFR 812.35 [23,24]; the EU and Korea have their own procedures [21,22]. Whether a given software change calls for submission, approval, documentation, or another action depends on these provisions and on the investigation. Two FDA documents have a narrower scope: the draft guidance on prescription drug use-related software concerns outputs that form part of drug labeling, and the guidance on predetermined change control plans concerns marketing submissions for specified AI-enabled device software functions [16,32].

Academic frameworks add detail on how measurements and interventions should be described. They also separate traceability from comparability: a recorded software version makes a change traceable, whereas comparable measurement across versions has to be supported by validation evidence. Verification, analytical validation, clinical validation, and the selection of reference measures each address a different question [33,34,35]. CONSORT-EHEALTH, the Template for Intervention Description and Replication (TIDieR), the mobile health evidence reporting and assessment (mERA) checklist, and TIDieR-telehealth cover intervention content, delivery, modification, and infrastructure [36,37,38,39]. Publications on bring-your-own-device studies and metadata describe the hardware, operating system, configuration, and processing information needed to interpret digital records [40,41]. The Electronic Clinical Outcome Assessment (eCOA) Consortium has discussed practical considerations in implementing sensor-based technologies [42]. An analysis of EMA regulatory procedures reports requests for evidence of equivalence between application versions and for recording and accounting for concurrent treatment when interpreting digital endpoints [43]. These examples describe advice given in the assessed procedures rather than requirements applicable to every digital intervention.

### 3.2. Reported Software Events and the Functions Affected

CureApp compared a therapeutic application, clinician software, and a home carbon monoxide (CO) checker with a minimal control application during standard smoking-cessation care [2]. Of 584 randomized participants, 572 formed the full analysis set (285 intervention, 287 control). Of these, 454 received varenicline, 114 nicotine patches, and four neither drug. Abstinence was verified by clinic CO measurement, separately from the intervention’s home checker. PMDA’s trial outline listed malfunctions and adverse events among the secondary endpoints [7]. PMDA recorded 85 malfunctions in 71 of 293 intervention participants (24.2%) and 44 in 43 of 291 controls (14.8%), a descriptive difference of 9.5 percentage points in participant proportions [7]. These counts establish reported malfunctions, not the number of participants whose therapeutic exposure or endpoint observations were altered. No malfunction was judged to have affected health, but interrupted sessions and lost endpoint observations were not quantified. Inability to install the app appeared among examples of reasons for discontinuation without a separate count [7]. The 12 app nonusers reported in the main article therefore cannot all be attributed to technical failure [2].

In Spark, the treatment itself changed. The report identified versions 2.1 and 2.2, their periods of use, and the content, interface, reward, and notification features added. Forty-one intervention participants received version 2.1 and 39 received version 2.2. The authors stated that the design, comparator, and outcome measures were unchanged and described the plan to combine versions. Version was included as a predictor in multiple imputation and as a fixed effect in outcome models, and no significant association between version and missingness was reported [44]. The accessible report did not provide a separate treatment-effect estimate for each version or an estimated treatment-by-version interaction.

In Quitting Matters, incomplete recording affected outcome measurement. The trial compared Learn to Quit-HIV with QuitGuide, with nicotine patches supplied in both groups. Engagement and usability were primary outcomes, and abstinence was secondary [45]. After an upgrade, engagement markers were incomplete for seven intervention participants, and the report stated that 32% of device interactions were lost. These figures use different units and were not combined into an event proportion. Partial markers were retained in the count analyses. This preserved recorded interactions but did not quantify all unrecorded activity. The report did not attribute missing abstinence observations to the software upgrade [45].

In Building Emotional Awareness and Mental Health (BEAM), an app-based mental health and parenting program, problems affected both access to treatment and the recording of engagement. The report described login problems and videos that did not play on some devices; in addition, the analytics configuration missed required usage variables, and a data-retention setting caused engagement data to be lost. When video playback could not be repaired during the trial, the team provided video links through a private YouTube channel. Attendance and forum participation were available from separate systems, and other treatment was recorded at enrollment but not during the trial [46].

The three-arm virtual reality trial for low back pain compared an active program (DTxP) with a sham program and standard care. Standard-care participants could later be rerandomized [47]. Deficiencies were actively elicited by telephone. Seven active-arm participants reported 20 deficiencies, and six sham-arm participants reported eight, including connection, program, controller, and audio problems. These involved the digital delivery systems, but the number of sessions lost from the 30-session program was not reported. No participant proportion was calculated because the report gave no explicit deficiency denominator and the active-arm intention-to-treat and safety counts differed (14 and 12). The reported deficiencies were not all established software-code defects.

The FDA recall of reSET-O concerned a therapeutic rule governing access to potential contingency-management rewards after positive urine test results [48]. The recall record attributed the problem to software design and reported an affected interval from 10 August 2020 to 30 April 2021, a corrective release on 30 April 2021, and 3370 units in commerce. Distributed units are not a denominator of affected patients. Because effectiveness might have been reduced, clinicians were encouraged to review affected patients and provide any treatment or counseling needed. The notice describes a correction and recommended follow-up care, but it does not establish that medication changed or that the recommended care occurred. Table 2 separates documented events and responses from information not established by the available reports.

Sources are cited in the first column. The first four columns summarize reported facts and the functions involved (RQ1). The fifth identifies information not established by those reports that would help assess the intended comparison (RQ2). It does not imply that the original investigators collected no such information. Absence of a documented consequence does not establish absence of an effect. The selected examples are illustrative and do not represent event frequency across trials.

The responses addressed different questions, including reported health effects in CureApp, version changes in Spark, incomplete engagement records in Quitting Matters, access problems in BEAM, device-deficiency ascertainment in the virtual reality trial, and a faulty reward rule in reSET-O [7,44,45,46,47,48]. The records provided event counts, descriptions, corrective actions, or analyses of the trial as conducted. They did not provide an estimate that isolated the causal change in clinical efficacy attributable to the particular event. This is a limitation of the event-linked evidence in these selected cases, not a claim that the trials lacked overall efficacy results.

### 3.3. Relationships Between Digital Functions and Medication Treatment

The trials placed the digital component differently relative to medication. In CureApp, NICO-THERA, and Quit Genius, digital treatment was added to smoking-cessation care that included pharmacotherapy [2,49,50], and TES and CT-152 were studied during buprenorphine treatment and antidepressant monotherapy, respectively [3,4,5]. Here, medication was the background against which the digital add-on was evaluated. In TeleDiab 1, TeleDiab 2, TELESAGE, and d-Nav, by contrast, the software supported insulin adjustment and so helped to determine medication use [51,52,53,54].

The trials also differed in whether medication was required or used by only some participants. Trials in schizophrenia, epilepsy, and depression after ketamine infusion specified medication treatment as part of the clinical setting [55,56,57], whereas other depression trials recorded medication use without requiring it [44,46,58,59]. Attention-deficit/hyperactivity disorder (ADHD) studies ranged from medication use in subsets to designs testing digital training with methylphenidate or during stable pharmacotherapy [60,61,62,63], and the ADAM-101 electroencephalography report described a subset of its trial [63,64]. Reports in pain, cardiometabolic disease, and other conditions provided further medication-use contexts [65,66,67,68,69,70,71,72,73,74,75].

Medication records differed in timing and detail. PEAR-004 assessed medication use separately from app engagement, ELAINE tabulated medication changes across intervals, and the Endocare trial reported use by day and arm [55,56,65]. Baseline use, follow-up information, and records linked to an event period were distinguished because assessing drug exposure during an event requires information linked to that period.

Supportive-care systems linked patient reports to clinical responses: electronic patient self-Reporting of Adverse-events: Patient Information and aDvice (eRAPID) operated during chemotherapy, and the CAPRI nurse navigation program combined remote monitoring with nurse navigation during oral anticancer treatment [76,77]. Although they are not therapeutic digital programs, they show how digital information can shape the clinical care patients receive.

Three relationships can therefore be distinguished: a digital add-on to background pharmacotherapy, software that guides medication use, and digital support for clinical care. Each requires different medication information when an event is assessed. These reports were used to characterize the relationships, not to estimate event effects.

## 4. Discussion

### 4.1. RQ1: Identifying the Functions Affected by Software Events

**Figure 2:**
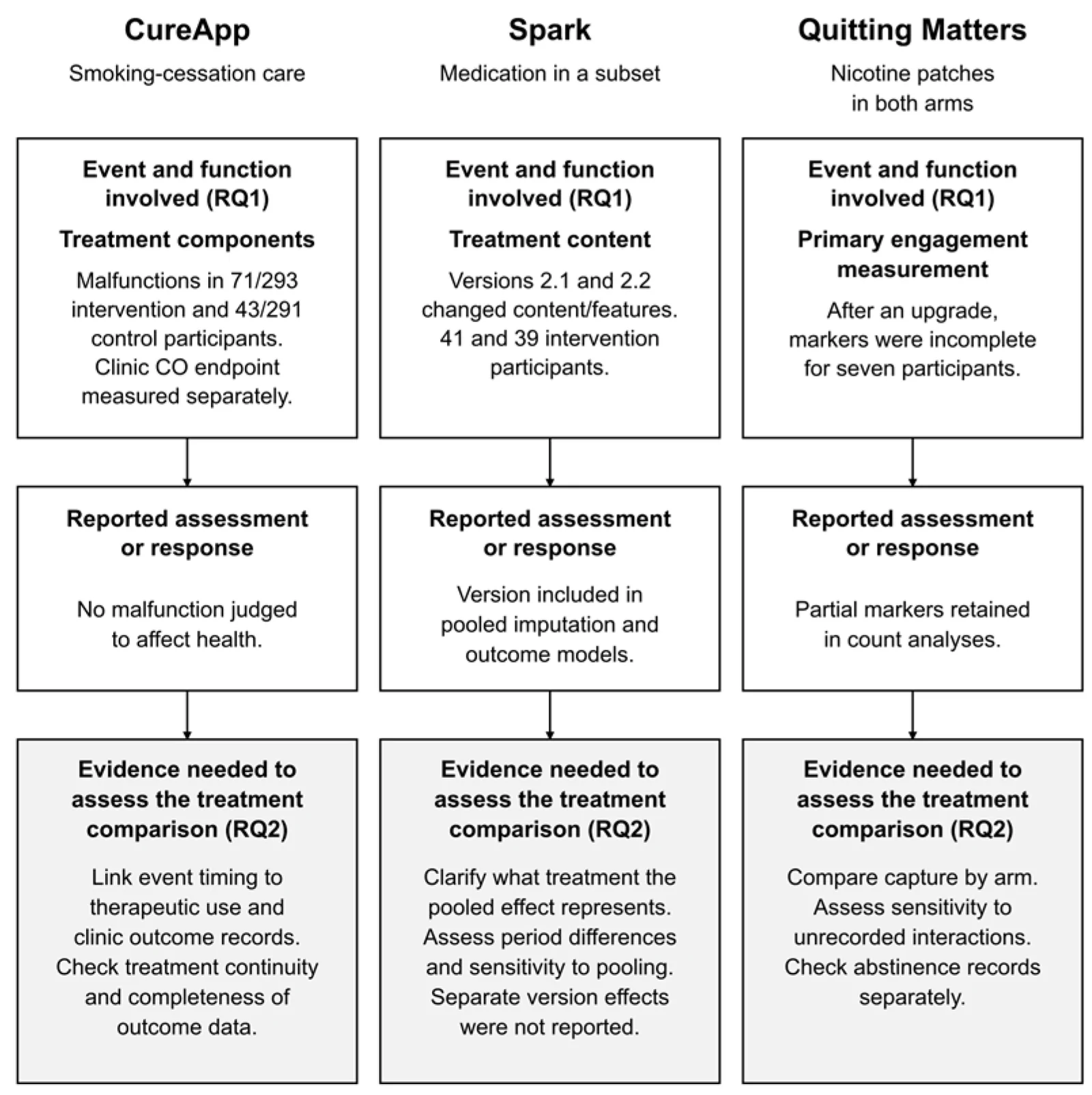
Linking software events and functions involved in appraisal of the intended treatment comparison. The diagram synthesizes evidence from the CureApp trial and PMDA assessment [2,7], Spark [44], and Quitting Matters [45]. The top row describes the event and function involved (RQ1), and the middle row summarizes the reported assessment or response. The shaded bottom row identifies evidence needed to assess the intended treatment comparison (RQ2). It represents the researchers’ appraisal, not a response reported by the original investigators. An event may affect more than one function. Medication use and replacement care should also be assessed when affected. The appraisal questions are not a validated regulatory decision tool and do not prescribe a regulatory decision or an estimand strategy.

The selected cases show that the event label does not identify its clinical consequence. CureApp reported malfunctions, Spark changed therapeutic content, and Quitting Matters lost engagement records. These events raise different questions about what treatment was delivered and what was measured. The first appraisal task is therefore to identify the function and the participants, trial groups, devices, and periods involved. Clinical importance depends on the extent to which the event altered that function, not only on its technical description or whether it was repaired. Table 2 and Figure 2 connect these observations to the information needed for the second research question.

CureApp illustrates the distinction between a malfunction and a demonstrated effect on treatment or measurement. The home CO checker formed part of the intervention, whereas clinic CO measurement supported the abstinence endpoint [2,7]. A problem in one pathway does not establish a problem in the other. PMDA’s finding that no malfunction affected health addresses safety. To assess treatment continuity and endpoint completeness, investigators would also need the malfunction type and timing linked to app access, completed therapeutic activities, and clinic outcome records. The difference in reported malfunction proportions between groups does not by itself establish differential exposure loss or biased abstinence measurement.

One event may affect several functions, which should be traced separately and then considered together. In BEAM, login and video problems affected access, whereas analytics omissions and the retention setting affected engagement records [46]. The team supplied replacement video links through a private YouTube channel when the playback problem could not be repaired during the trial [46]. This documents a workaround, but its availability does not establish who watched the replacement videos or how much interrupted treatment it restored. Attendance and forum records describe additional participation but cannot reconstruct every app session. In practice, investigators can first identify the treatment and measurement pathways affected during the same interval, then examine their consequences for the comparison. If medication decisions or replacement care also changed, those pathways need the same event-linked assessment. This avoids equating missing usage records with nonuse or treating restored access as proof that outcome recording also recovered. Recorded use also differs from therapeutic dose. A recent viewpoint distinguishes platform exposure from engagement, skill acquisition, and use of skills outside an application [78]. Session counts alone therefore do not establish how much therapeutic benefit was lost.

Medication changes the appraisal according to its role. When digital treatment is added to background pharmacotherapy, the effect of interest concerns the digital add-on under the specified medication and comparator-care conditions. CureApp, TES, and MIRAI illustrate this relationship [2,3,4,5], as does the PEAR-008 versus reSET-O protocol during buprenorphine treatment [79]. A software event need not alter pharmacotherapy, but any associated drug change or additional care can change the combination actually received. In trials with medication use in only a subset, baseline co-use is insufficient to characterize drug exposure during an event. Follow-up records, such as those described for PEAR-004, ELAINE, and Endocare, would need to be linked to the affected interval [55,56,65].

The regimen can also limit the efficacy claim independently of a technical event. In the varenicline subgroup of CureApp, PMDA reported continuous abstinence at weeks 9–24 of 67.0% (152/227) with the intervention and 50.7% (115/227) with the control. During the authorization review, PMDA accepted a revised specification identifying varenicline as the medication used with the algorithm [7]. This was an authorization decision, not a trial software update. It illustrates why the medication regimen defining the efficacy claim must remain distinct from any consequence attributed to a malfunction.

When software guides medication use, availability and accuracy are part of the drug-treatment pathway. The Diabeo system evaluated in the TeleDiab 1 and TELESAGE trials, and the d-Nav insulin guidance system, supported insulin dose adjustment [51,53,54]. An event affecting that function calls for assessment of the recommendations available, the doses actually taken, and any clinician instructions replacing the software. New recommendations produced by an unchanged algorithm are normal operation and are not themselves software changes. Supportive-care systems provide another pathway. eRAPID measured chemotherapy delays, dose reductions, and drug changes without finding between-group differences [76]. CAPRI combined digital monitoring with nurse navigation and increased relative dose intensity from 89.4% to 93.4% in 559 evaluable patients [77]. These are results of the programs as delivered, not estimates of software-event effects, but they show why medication delivery and the clinical response to digital information should be considered separately.

Replacement care may preserve access to treatment while changing the services that define the comparison. The reSET-O notice advised clinicians to review treatment and counseling needs after correcting the reward rule [48]. It did not report whether that advice was followed. In a trial, relevant information would include the replacement care actually provided, its timing, and what the comparator group received. Implementation reports from BD4QoL and eRAPID provide related context on digital systems within care pathways [80,81]. Such context supports appraisal of the pathway, without establishing that a particular malfunction changed drug exposure or efficacy.

### 4.2. RQ2: Assessing Whether the Intended Treatment Comparison Remains Supported

The second question concerns what treatment effect the available data can estimate. ICH E9(R1) expresses this through the estimand, which specifies the population, treatments, outcome, population-level summary, and handling of events after treatment starts [10]. In plain terms, an analysis may ask about assignment to the program as actually delivered, including interruptions, or about a clearly defined situation without a particular interruption. An event that changes treatment received may be an intercurrent event. An event that disrupts observation may instead create missing or unreliable data, and one event can do both. The technical cause alone does not determine the appropriate strategy.

Spark illustrates the distinction between adjusting for version and estimating each version’s effect. The trial planned to combine versions and included version in its imputation and outcome models [44]. The pooled estimate therefore concerns the program as delivered across both versions and periods. A version covariate does not by itself provide separate randomized treatment comparisons for versions 2.1 and 2.2 or establish that they have equal effects. Because versions were used in different calendar periods, version may also reflect differences in recruitment, co-treatment, or study circumstances. Where the data and sample size permit, treatment comparisons within periods using concurrent controls, a treatment-by-period interaction, and sensitivity analyses of pooling could assess dependence on the version mix. Such estimates need uncertainty intervals and do not constitute randomized comparisons between software versions [14,44].

Quitting Matters shows why statistical handling of observed counts does not establish complete capture. Learn to Quit-HIV used Google Analytics, whereas QuitGuide data were transferred through the National Cancer Institute [45]. Retaining partial markers in the zero-inflated negative-binomial count analyses preserved available information, but those models do not automatically correct interactions lost from one recording system. If capture loss differs between groups, identical actual use could generate different observed counts. Missing activity should not be treated as zero without justification. Event timing, affected participants, recording coverage in both groups, and independent records of use would help distinguish lower engagement from under-recording. Analyses of comparable recording periods or sensitivity analyses under explicit assumptions about the unrecorded interactions could show how the primary engagement comparison depends on capture completeness [14,45].

Differential capture can also misclassify exposure or outcomes. Incomplete logs may classify a participant as a nonuser despite treatment use. A changed scoring rule may classify the same underlying observation differently across versions or groups. These are possible mechanisms, not demonstrated biases in every case. Investigators should examine whether ascertainment and processing differed by arm, device, site, or period and whether affected observations can be recovered or reprocessed. Abstinence in Quitting Matters and clinic CO measurement in CureApp used pathways separate from the affected engagement or home-device functions [2,7,45]. This separation limits what can be inferred from the technical event, but does not replace checking the completeness of those clinical outcomes.

Statistical handling should follow the affected outcome and the estimand. Under a treatment-policy strategy, outcomes remain relevant after treatment disruption, so follow-up should continue. A hypothetical strategy requires a defined alternative treatment condition and assumptions about both digital and medication treatment. Excluding participants with technical problems or restricting analysis to application users can change the comparison and introduce selection bias [10,82]. For missing clinical outcomes, likelihood-based analysis or multiple imputation may be appropriate when their assumptions are defensible. Models should consider observed predictors of missingness and outcome, potentially including baseline outcome, trial arm, site, version, and period. The variables and complexity must be supported by the design and data [14,82,83].

Technical causation does not establish that data are missing completely at random. Device characteristics, deployment period, or treatment stage may relate to both missingness and outcome. Spark’s reported lack of a significant association between version and missingness does not prove a missing-at-random assumption [44]. Sensitivity analyses can vary assumptions about unobserved outcomes, for example by applying plausible departures from an imputation model and identifying when the clinical conclusion changes. For partially captured engagement counts, assumptions must concern the lost interactions and recording coverage rather than simply borrowing an outcome-imputation model. Analyses should distinguish unavailable, corrupted, and verified observations, document recovery or exclusion rules, and compare conclusions under defensible alternatives [14,34,82,83]. For sensor-derived outcomes, Di et al. distinguish gaps in high-frequency measurements from missing daily summaries and describe how data-sufficiency thresholds affect the observations included in endpoint analyses [83]. Their recommendations support specifying the level of missingness and testing data-sufficiency and missing-data assumptions in sensitivity analyses [83].

An event affecting infrastructure can involve several participants at once, at different treatment stages. Calendar period, site, device, and time since randomization may therefore matter. The CTN-0100 experience involved loss of digital treatment supply and, separately, a medication supply problem, raising questions about interrupted exposure and concurrent treatment comparisons [84]. Supply withdrawal differs from a software failure, but the example shows why the remaining treatment conditions and concurrent controls matter. Similarly, PD STAT reported loss of exploratory digital assessment data after an update, whereas eSMART reported an interruption of remote symptom support during chemotherapy [85,86]. These contextual systems illustrate different pathways, and neither establishes an event-attributable efficacy effect for a therapeutic digital add-on.

Technical recovery and measurement comparability require separate evidence. Version control should identify deployed builds and configurations, while audit trails preserve changes to records and their timing. Predefined change-management procedures can specify impact assessment, testing, deployment, rollback, and documentation responsibilities. Historical builds, configuration files, processing rules, and raw records should be preserved where feasible and consistent with applicable requirements so that earlier observations can be interpreted or reproduced. Archiving a historical version does not mean continuing to expose participants to an unsafe version [8,9,11,27,28]. A restored connection resumes transmission but does not recover every lost observation. Verification, analytical validation, and clinical validation address different aspects of whether a measure remains fit for its intended use [33,34,35]. The FDA paper on digitally derived measures (August 2026) provides further methodological context rather than a separate binding requirement [87].

When an event could affect outcome classification, independent or blinded assessment may reduce discretionary bias. Where feasible, assessors can apply predefined rules to retained source records without knowing treatment allocation or the result favored by a particular classification. The same rules should apply across groups and periods, and disagreements and adjudication decisions should be documented. This can strengthen the assessment of ambiguous or corrupted observations. It cannot recreate unavailable raw data, demonstrate equivalence between versions, or compensate for a systematically missing outcome. Spark used separate clinical review and masking of the treatment group where possible for safety classification [44]. That example concerns safety and does not establish independent adjudication of corrupted efficacy endpoints. The options described here are consistent with protecting trial-data reliability, not an adjudication procedure shown to have been used in all reviewed cases [9,10,18].

Clinical interpretation and regulatory action are related but separate judgments. The function-based appraisal is an interpretive framework derived from the selected evidence and existing principles. This appraisal creates no additional regulatory obligations. Whether a change requires an amendment, notification, approval, or documentation depends on the applicable drug or device provisions [21,22,23,24]. Agency guidance and methodological recommendations should not be presented as equivalent to binding law. Lifecycle literature discusses evidence generation as products evolve [12,88,89,90], and Colloud et al. describe how intended use can bring digital tools within medicinal-product or medical-device frameworks [91]. The MFDS change-management-plan guidance has the specific scope of AI-enabled digital medical devices [31]. A new efficacy trial is not necessary for every update, but the evidence supporting continued applicability of the original result must match the changed function [8,9,27,30].

Compared with reviews of digital endpoints and software adherence [12,13,14,15], this synthesis links documented responses to the medication pathway and the comparison that remains supported. Future trials could record software versions, deployment and event dates, affected participants and groups, interruptions, replacement care, medication use, and outcome-data completeness prospectively. Linking these records at participant level would permit analyses of event periods and sensitivity to missingness or version changes. Investigators could then report which conclusion is supported, which assumptions it depends on, and which uncertainty cannot be resolved. These proposals make existing reporting and appraisal principles more usable, but their ability to improve clinical outcomes has not been established by this review [8,9,10,11,36,37,38,39,40,41,42].

### 4.3. Limitations

This review used a single bibliographic database and purposive source selection. One author screened all records, and a second reviewed the candidate records and confirmed the selection decisions. Selection bias cannot be excluded, and relevant event reports may have been missed. The cases were chosen to illustrate different functions, event types, and responses, not to represent the occurrence or typical consequences of software events. The evidence spans trials requiring medication, trials with medication use in a subset, medication-guidance software, and contextual supportive-care or measurement systems. Not all evaluated a licensed drug with a digital therapeutic product. The review did not estimate event frequency, compare reporting quality, or pool efficacy data.

Available information differed across trial articles, regulatory assessments, implementation records, and postmarketing notices. Main-article text was available for all 67 publications retained from PubMed, but separate supplements, image-only material, and unpublished technical logs were not always accessible. CureApp’s malfunctions were reported by PMDA rather than the main trial article. Absence of a documented consequence therefore does not show absence of impact, and a response not described in public reports should not be assumed inadequate.

Medication use and outcome observations were not consistently linked to event periods. The selected reports therefore cannot isolate how much clinical efficacy was lost or gained because of an event. Severity could not be graded on a common scale, and conditional analyses discussed here were not performed on participant-level data. The appraisal questions in Supplementary Method S5 have not been validated as a decision instrument. Prospective evaluation is needed to establish when these questions materially change a clinical or regulatory conclusion.

## 5. Conclusions

Software events require assessment of both intervention integrity and data integrity. For RQ1, investigators need to establish whether an event affected digital treatment delivery, outcome measurement, medication use, or replacement care, allowing more than one function to be involved. For RQ2, they need to determine whether the retained data and analysis still address the intended treatment comparison. The selected cases show why a safety assessment, technical repair, or model adjustment answers only part of this question. Prospective links between versions, event timing, treatment exposure, medication and replacement care, and outcome records can make the comparison assessable and support sensitivity analyses. These measures strengthen the basis for interpreting benefit. The review does not establish that they improve clinical efficacy or that any single analytical strategy is appropriate for every event.

## Figures and Tables

**Table 1:** Selected public guidance and legal provisions for software events in clinical trials.

Document, Version, and Status	Scope and Provisions Relevant to the Review Questions
FDA DHT, December 2023 [8] Final guidance	Remote data acquisition in clinical investigations. Covers the assessment of updates, comparison of measurements across versions, and analysis of relevant differences.
EMA computerised systems, March 2023 [9] Final guideline	Computerized systems in trials of investigational medicinal products. Covers validation, change control, audit trails, access control, and data integrity.
ICH E9(R1), November 2019 [10] Final Step 4 guideline	Definition and estimation of treatment effects. Provides a framework for specifying the treatment effect of interest and strategies for handling intercurrent events.
ICH E6(R3), Principles/Annex 1 and Annex 2 [18,19] Final Step 4 guidelines, January 2025 (corrected October 2025) and June 2026	Good clinical practice. Calls for fit-for-purpose systems, data governance, and reliable, traceable trial records.
MFDS DTx trial design, October 2025 [20] Guidance (Guide 1391-02)	Digital therapeutic trials in Korea. Discusses comparator selection; its frequently asked questions explain which changes require amendment approval under the applicable regulation.
MDCG 2021-6 Rev. 1, December 2023 [21] Guidance	EU medical-device investigations. Explains procedures for changes to clinical investigations, including substantial modifications.
MFDS Notice 2025-29, April 2025 [22] Delegated regulation	Digital-device investigations in Korea. Sets out provisions for approval, trial conduct, and amendments.
21 CFR 812.35 and 312.30 [23,24] US regulations	US device and drug investigations. Specify separate procedures, with defined exceptions, for changes to investigations and for protocol amendments.
MFDS digital data collection 24 December 2025 [11] Applicant guidance	Digital data collection in drug trials. Covers software version policies, the suitability and impact of updates, proportionate performance checks, and user training.
MFDS nicotine-use-disorder protocols 10 September 2026 [25] Applicant guidance	Protocols for digital therapeutics in nicotine use disorder. Describes software versions and functions, communication arrangements where applicable, treatment instructions, and planned use schedules.
MFDS digital-device trials 30 April 2026 [26] Applicant guidance	Digital-device investigations and off-site procedures. Covers remotely collected endpoint data, measurement differences, missing data, and procedures for reporting and recovering from network failures, device errors, and data loss.
MFDS software validation 28 July 2026 [27] Applicant guidance	Medical-device software validation. Recommends change-impact analysis and risk reassessment, with verification and/or validation according to the change and its impact on the device or software system.

**Table 2:** Reported software changes and technical problems, responses, and unresolved information.

Source and Setting	Documented Event	Function Involved (RQ1)	Reported Assessment or Response	Information not Established by Available Reports (RQ2)
CureApp [2,7] Trial and PMDA assessment	85 malfunctions in 71/293 active and 44 in 43/291 control participants	Treatment components. Clinic endpoint measured separately.	No malfunction judged to have affected health.	Event-linked missed treatment and lost endpoint observations were not quantified.
Spark [44] Trial with medication use in a subset	Versions 2.1 and 2.2 differed in content and features (41 and 39 active participants).	Digital treatment content	Pooling planned. Version included in imputation and outcome models.	Separate treatment effects for each version and dependence of the pooled effect on the version mix.
Quitting Matters [45] Nicotine patches in both arms	Upgrade left engagement markers incomplete for seven participants.	Primary engagement measurement	Partial markers retained in count analyses.	Full recovery of lost interactions and the effect of unequal capture on the engagement comparison.
BEAM [46] Trial with medication use in a subset	Login and video problems. Incomplete or lost engagement data.	Treatment access and engagement recording	Email troubleshooting and video links through a private YouTube channel. Separate attendance and forum records.	Use of the replacement video links, extent of compensated treatment, and event-linked medication changes.
Virtual reality pain trial [47] Concomitant analgesics	20 deficiencies in seven active and eight in six sham participants.	Digital delivery systems in both arms	Deficiencies elicited by telephone and reported.	Sessions lost and their consequences for treatment exposure or the clinical comparison.
reSET-O [48] Postmarketing recall	Reward-rule error from 10 August 2020 to 30 April 2021.	Therapeutic reward rule and advised follow-up care	Corrective release. Clinicians advised to review treatment and counseling needs.	Actual follow-up care, medication changes, and clinical consequences of the event.

## Data Availability

The search queries, source-selection methods, and inventory of retained sources are provided in the Supplementary Materials. No individual-level participant data were collected.

## Supplementary Materials

The following supporting information can be downloaded at https://www.mdpi.com/article/10.3390/healthcare14213604/s1: Supplementary File S1: Supplementary Methods (The Supplementary Methods describe the PubMed search components, the official-source searches and document selection, the source-selection procedure and criteria, the extraction and verification methods, and the case-appraisal method). Supplementary Method S1. PubMed search strategy. Supplementary Method S2. Official-source searches and document selection. Supplementary Method S3. Review design and source selection. Supplementary Method S4. Extraction and source verification. Supplementary Method S5. Case appraisal. Supplementary File S2: Data list (The Supplementary Data list the 67 academic publications retained from PubMed and their reference numbers).

## Abbreviations

ADHD	Attention-deficit/hyperactivity disorder
AI	Artificial intelligence
BEAM	Building Emotional Awareness and Mental Health
CAPRI	name of the nurse navigation and remote monitoring program for oral anticancer treatment
CFR	Code of Federal Regulations
CO	Carbon monoxide
CONSORT	Consolidated Standards of Reporting Trials
DHT	Digital health technology
DTx	Digital therapeutics
DTxP	Label for the active digital therapeutic pain program
eCOA	electronic clinical outcome assessment
EMA	European Medicines Agency
eRAPID	electronic patient self-Reporting of Adverse-events: Patient Information and aDvice
eSMART	electronic Symptom Management using the Advanced Symptom Management System Remote Technology
EU	European Union
FDA	US Food and Drug Administration
ICH	International Council for Harmonisation
MDCG	Medical Device Coordination Group
mERA	Mobile health evidence reporting and assessment
MFDS	Ministry of Food and Drug Safety
NCBI	National Center for Biotechnology Information
NCI	National Cancer Institute
PMDA	Pharmaceuticals and Medical Devices Agency
TES	Therapeutic Education System
TIDieR	Template for Intervention Description and Replication
