# Supplementary material for: Software Changes and Technical Failures in Clinical Trials of Digital Interventions Used Alongside Pharmacotherapy: Narrative Review of Regulatory Guidance and Published Evidence
Source: Healthcare (Basel). 2026 Oct 24;14(21):3604. doi: 10.3390/healthcare14213604 (PMC13762610; doi:10.3390/healthcare14213604)
Supplement: Supplementary file 1 [file healthcare-14-03604-s001.zip › Supplementary_Methods.pdf]

Supplementary Methods

Supplementary Method S1. PubMed search strategy

PubMed was searched using National Center for Biotechnology Information (NCBI) E-utilities on 8 to 10 September 2026 without publication-date or language filters. The search frame included topic, product-name and trial-name components, grouped into the three domains below. Domain yields were reconstructed from the archived search results. Records identified solely through DOI, exact-title or author-specific lookups were outside this frame. The clinical, software-event and methodological domains contained 528, 498 and 91 unique PMIDs, respectively. Deduplication across these domains yielded 1,093 records.

Clinical evidence in medication-based care

The results contained 528 unique PMIDs.

```
(("digital intervention"[tiab] OR "digital interventions"[tiab] OR "digital therapeutic"[tiab] OR "digital therapeutics"[tiab] OR "prescription drug use-related software"[tiab] OR PDURS[tiab] OR "software as a medical device"[tiab]) AND (pharmacotherapy[tiab] OR medication*[tiab] OR drug*[tiab] OR pharmaceutical*[tiab]) AND (review[pt] OR review[tiab] OR framework*[tiab] OR regulat*[tiab]))
OR
((Diabeo[tiab] OR TeleDiab[tiab] OR d-Nav[tiab] OR CAPRI[ti] OR eRAPID[ti] OR eSMART[ti]) AND (random*[tiab] OR trial[ti]))
OR
(("digital therapeutics"[tiab] OR "digital therapeutic"[tiab] OR "prescription digital"[tiab]) AND (adjunct*[tiab] OR pharmacotherap*[tiab] OR medication*[tiab] OR buprenorphine[tiab] OR varenicline[tiab] OR nicotine[tiab] OR antidepressant*[tiab]) AND (trial*[tiab] OR implementation[tiab] OR pilot[tiab]))
OR
((CureApp[tiab] OR "reSET-O"[tiab] OR "CT-152"[tiab] OR Rejoyn[tiab]) AND (trial*[tiab] OR implement*[tiab] OR pilot[tiab]))
```

Software events and outcome interpretation

The results contained 498 unique PMIDs.

```
(("digital health"[tiab] OR "digital therapeutic"[tiab] OR "digital therapeutics"[tiab] OR "software as a medical device"[tiab] OR PDURS[tiab] OR "mobile health"[tiab] OR "medication app"[tiab]) AND (trial*[tiab] OR investigat*[tiab]) AND ("software update"[tiab] OR "software updates"[tiab] OR "software change"[tiab] OR "software changes"[tiab] OR "algorithm change"[tiab] OR "algorithm changes"[tiab] OR "version control"[tiab] OR "technical failure"[tiab] OR "technical failures"[tiab] OR "technical issues"[tiab] OR outage*[tiab] OR malfunction*[tiab] OR "change control"[tiab] OR "software modification"[tiab]))
OR
(("digital health"[tiab] OR "digital endpoint"[tiab] OR "digital endpoints"[tiab] OR "digital outcome"[tiab] OR "digital outcomes"[tiab] OR "digitally derived"[tiab]) AND (trial*[tiab] OR "clinical investigation"[tiab] OR "clinical investigations"[tiab]) AND (estimand*[tiab] OR "intercurrent event"[tiab] OR "intercurrent events"[tiab] OR "missing data"[tiab] OR comparability[tiab] OR "data integrity"[tiab] OR regulat*[tiab] OR qualification[tiab]))
```

Methodological guidance

The results contained 91 unique PMIDs.

```
((CONSORT-EHEALTH[ti] OR CONSORT-AI[ti] OR SPIRIT-AI[ti] OR DECIDE-AI[ti] OR mERA[ti] OR (intervention description[ti] AND replication[ti])) AND (guideline*[ti] OR reporting[ti] OR checklist[ti] OR evaluation[ti]))
```

```
OR
((digital[ti] OR sensor*[ti] OR wearable*[ti] OR biometric[ti]) AND (verification[ti] OR
validation[ti] OR usability[ti] OR fit-for-purpose[ti] OR novel endpoint*[ti]) AND
(framework[ti] OR recommendation*[ti] OR V3[tiab]) AND (clinical[tiab] OR measurement*[tiab]))
OR
((missing data[ti] OR missing outcome*[ti]) AND (clinical trial*[ti] OR randomized trial*[ti]
OR intention to treat[ti]) AND (prevention[ti] OR strategy[ti] OR sensitivity[ti] OR
controlled multiple imputation[ti]))
```

Supplementary Method S2. Official-source searches and document selection

The researchers searched official domains and followed document cross-references through 10 September 2026. Sources were fda.gov, ecfr.gov, govinfo.gov, ema.europa.eu, health.ec.europa.eu, ich.org, mfds.go.kr, law.go.kr and pmda.go.jp. The researchers combined document or topic terms with the relevant domain and checked documents against issuing-agency or legal-source pages.

Search topics included prescription drug use-related software, digital data acquisition, computerised trial systems, electronic records, software validation and change control, digitally derived measures, clinical-investigation modifications, and ICH E9(R1) and E6(R3). Korean searches covered digital therapeutic trial design and protocols, digitally enabled trial data collection, digital-device investigations, authorization, change-management plans, software validation and management-system certification. Product searches used CureApp SC and the PMDA assessment, and reSET-O and FDA recall Z-1926-2021.

Selection considered relevance to the review question, issuing authority and document version. Final guidance, drafts, legislation, regulations and agency discussion papers were distinguished, and linked language versions were counted once. Among seven MFDS indication-specific protocol guides issued on 10 September 2026, the nicotine-use-disorder guide was retained for its direct connection to the cessation example. The other indication guides, an institutional standard operating procedure guide and quality-system frequently asked questions were checked but not counted as independent retained sources. English renderings of Korean titles were labeled as author translations where no official English title was verified.

The 22 retained public documents comprised seven US sources [8,16,23,24,28,32,87], two EU sources [9,21], three ICH documents [10,18,19] and ten Korean sources [11,17,20,22,25-27,29-31]. The PMDA CureApp assessment [7] and FDA reSET-O recall [48] were counted separately as two product-specific records. Manuscript Table 1 summarizes selected provisions relevant to the review questions. This focused selection does not establish exhaustive coverage of all official guidance.

Supplementary Method S3. Review design and source selection

This focused narrative review combines public official documents with published methodological and clinical literature. The synthesis examines how software changes and technical failures affect treatment delivery, outcome measurement and interpretation in trials involving pharmacotherapy. It compares the event observed, the assessment or response reported, and the evidence needed to judge remaining uncertainty. Clinical reports were not used to estimate software-event frequency or calculate reporting-quality scores. The narrative design supports interpretation across heterogeneous source types. It is not intended as exhaustive evidence mapping or as a representative sample of events.

The included PubMed search frame contained 1,093 unique identifiers. I.H. reviewed the titles and available abstracts of these records and consulted full texts when needed to assess relevance. I.H. selected 75 records for detailed examination. S.-W.L. reviewed these records and confirmed their selection for detailed examination. The remaining 1,018 records were not selected for detailed examination.

Prioritization considered three types of contribution. Event reports could describe an actual software change or technical failure and its assessment or handling. Clinical reports could explain the digital function, medication pathway and comparator care. Methodological or regulatory sources could provide principles relevant to evaluating the event or interpreting the treatment comparison. A broad methodological topic alone did not establish a distinct contribution. Its relevance depended on how it informed a question about treatment delivery, outcome measurement or interpretation.

Source assessment considered six criteria. C01 concerned public availability, identifiable authorship or issuing authority, and a traceable publication or document version. C02 concerned relevance to software events, treatment continuity, validation, endpoint comparability, data integrity, estimands, missing data, or related analysis and reporting. C03 concerned the contribution and scope of the source. Direct clinical illustrations needed an identifiable digital function and medication pathway. Supporting methodological sources could address broader settings if their principles were relevant to the review question. C04 concerned accessible English or Korean content sufficient to assess the information used. C05 concerned the issuing authority and document version, with current final guidance distinguished from identified drafts and linked versions or translations. C06 concerned a distinct evidentiary contribution. Promotional authority claims, unrelated generic accounts and redundant versions without an analytical purpose were not retained.

The principal examples provide publicly documented events and enough information to distinguish the event from its assessment or response. CureApp documents an official health-impact assessment during medication-based smoking-cessation care. Spark describes therapeutic-content changes and analyses incorporating software version. Quitting Matters describes incomplete engagement records affecting a primary outcome during nicotine-patch treatment. These examples support comparison of different clinical and analytical questions. They do not establish the frequency or typical consequences of software events.

Clinical reports without documented software events were used to explain background medication, medication guidance and supportive care within the treatment comparison. Insulin-guidance studies describe a direct dosing function. eRAPID and CAPRI describe clinical responses and medication delivery within supportive care. Implementation studies and postmarketing records were interpreted in their own settings. Related reports could contribute different information about a study, whereas sources that repeated an already represented contribution without additional relevant information were not retained.

Detailed examination of the 75 candidate sources resulted in 67 retained academic publications and eight nonretained publications. HERB-DH1 (PMID 34455443) did not provide a direct medication-linked clinical illustration because its primary comparison excluded antihypertensive medication. The reSET-O feasibility report (PMID 35333189) provided overlapping treatment-context information. The formative implementation evaluation (PMID 39676816) addressed broader implementation processes and was not used as an additional case in the focused comparison. CONSORT-AI, SPIRIT-AI, DECIDE-AI and the CONSORT-AI concordance study (PMIDs 32908283, 32908284, 35584845 and 38388497) focused on AI-specific intervention reporting and did not contribute to the event appraisal used here. The Mobgap software report (PMID 42451535) addressed gait-algorithm implementation and validation without contributing to the medication-linked event comparison. Relevant full-text content was available for all 67 retained academic publications.

All 67 retained academic publications were among the 1,093 records in the included search frame. Supplementary Data provides a full reference for each retained publication and its corresponding

manuscript reference number. All 67 publications are cited in the manuscript. The official-document component comprised 22 regulatory, agency, legal or ICH documents and two product-specific records. The searches and source assessment were focused on the review question and do not establish exhaustive coverage of all relevant literature.

Supplementary Method S4. Extraction and source verification

The clinical sources describe patient-facing therapeutic functions and medication context. Medication could be required by the study design or documented among enrolled participants. Baseline use was distinguished from treatment information collected during follow-up. Extraction recorded the digital function, medication evidence, comparator and source location for the clinical reports. Related publications remained individually identifiable.

For each case, extraction recorded the affected function, the event described, the assessment or response reported, and the information relevant to treatment delivery, outcome observations, and their analysis. Journal XML or BioC text, tables, and figure captions supported source navigation. Access to supplementary files and image-only content varied and was not assumed. Actual events were distinguished from hypothetical risks, routine software descriptions, and events cited from other studies. No event-frequency estimate or negative-event classification was derived from the absence of an event description in accessible text. The Spark main article and its two supplementary appendices were examined for version-specific treatment effects and interaction analyses. The Quitting Matters main article was examined for the handling of partially recorded engagement markers, separately from its abstinence analyses.

Medication information was connected to an event period only when the source provided that link. Baseline medication counts alone do not establish drug exposure during an event. Regulatory product assessments, implementation findings, and postmarketing records retain their own source settings.

The main-manuscript references were checked against bibliographic or issuing-source records. Academic metadata were compared with PubMed and Crossref where available, and with journal or repository records. Official references were checked against agency or legal sources. Source titles, authors, dates, identifiers and available publication details were retained without inventing unassigned volume or issue information. Bibliographic verification is distinct from verification of the scientific claim supported by a citation.

CureApp claims used PMDA's official English translation [7]. PMDA states that the Japanese original prevails. The authors did not independently verify the Japanese original.

I.H. conducted the title and abstract review described in Supplementary Method 3 and reviewed the retained evidence. S.-W.L. confirmed the records selected for detailed examination. The authors reviewed and edited the manuscript and take responsibility for its content.

Supplementary Method S5. Case appraisal

The researchers appraised each event in relation to the planned treatment comparison. RQ1 identified affected functions and RQ2 considered what the reported response resolved and what evidence was needed to judge remaining uncertainty. The questions below were not a validated checklist, regulatory decision instrument, or reporting-quality score.

Appraisal considered the affected function and configuration, the event and recovery dates, and the participants, trial groups, sites or devices involved. Counts were interpreted using the reported

denominator and ascertainment method, distinguishing people, sessions, records and distributed units. These details locate the event in the treatment pathway and relevant observation period [7-11,27,36,40-44,48]. Clinical importance was described qualitatively using the function, duration, extent, trial groups, treatment or measurement consequences, and recovery. No common severity score was assigned. Unreported consequences remained uncertain rather than being classified as minor.

Treatment appraisal concerned what digital treatment remained available or was delivered, including altered content, missed sessions and replacement care. Medication and comparator information mattered when dose guidance, drug exposure, adherence or additional care could change the comparison. Nonuse was distinguished from unavailable treatment or missing usage records. Usage time alone was not treated as a validated therapeutic dose [1,3,4,10,36-39,45,46,55,56,65,76-78]. Events could affect more than one function. Delivery, medication, replacement-care and observation pathways were considered separately before their combined implications for the comparison were appraised.

Measurement appraisal distinguished clinical endpoints from engagement and safety measures. Relevant evidence included missing, delayed, duplicated or overwritten observations, their timing and extent, and changes to eligibility or analysis-set membership. Recovery or reprocessing was considered alongside preservation of raw records and evidence of comparability across versions. Absence of harm did not establish complete outcome data [7-11,26,27,33-35]. Appraisal also considered whether recording or classification could differ between groups, versions, sites or periods. Independent or blinded assessment of retained records was discussed as a possible safeguard, not assumed to have occurred in every study.

Analytical appraisal considered the treatment represented by pooled versions, calendar-period differences, missing-data assumptions, relevant protocol or analysis-plan amendments and the evidence that could test remaining uncertainty. Comparable observation windows, period-specific comparisons and sensitivity analyses could help address these questions where feasible [10,14,44,82,83]. These are conditional appraisal questions, not a mandatory estimand strategy or new regulatory requirements. Applicable obligations for medication records, participant-level traceability, safety surveillance and change control continue to apply. Statistical options depend on the affected outcome and assumptions. Missing clinical outcomes may support likelihood-based analysis or multiple imputation when their assumptions are defensible, with sensitivity analyses for departures from those assumptions. Partially captured engagement counts require attention to recording coverage and cannot automatically be treated as zero use. These analyses were discussed conditionally and were not conducted on participant-level data in this review. Version histories, change-control records and prospective links between event dates, interruptions, medication, replacement care and outcome completeness were considered relevant to making those assumptions assessable.
